# Supplementary material for: Short report: Spatial distribution and growth of sheep farming in Brazilian Amazon
Source: PLoS One. 2022 Dec 1;17(12):e0278691. doi: 10.1371/journal.pone.0278691 (PMC9714815; doi:10.1371/journal.pone.0278691)
Supplement: Attachment — Submitted filename: Response to reviewers.docx [file pone.0278691.s001.docx]

**Response to reviewers**

**Journal Requirements:**

1. When submitting your revision, we need you to address these additional requirements.

Please ensure that your manuscript meets PLOS ONE's style requirements, including those for file naming.

Response: We have reviewed this and made the missing adjustments mainly in the tables.

2.Thank you for stating the following financial disclosure:

"This study received financial support for publication fee from Pró-Reitoria de Pesquisa e Pós-Graduação (PROPESP/UFPA). Also, the first author received scholarship from the Coordenação de Aperfeiçoamento de Pessoal de Nível Superior (CAPES) – Brasil (Finance Code 001).” Please state what role the funders took in the study. If the funders had no role, please state: "The funders had no role in study design, data collection and analysis, decision to publish, or preparation of the manuscript.". If this statement is not correct you must amend it as needed. Please include this amended Role of Funder statement in your cover letter; we will change the online submission form on your behalf.

Response: We have included the amended Role of Funder statement in the cover latter.

3.In your Data Availability statement, you have not specified where the minimal data set underlying the results described in your manuscript can be found. PLOS defines a study's minimal data set as the underlying data used to reach the conclusions drawn in the manuscript and any additional data required to replicate the reported study findings in their entirety. All PLOS journals require that the minimal data set be made fully available. For more information about our data policy, please see http://journals.plos.org/plosone/s/data-availability.

"Upon re-submitting your revised manuscript, please upload your study’s minimal underlying data set as either Supporting Information files or to a stable, public repository and include the relevant URLs, DOIs, or accession numbers within your revised cover letter. For a list of acceptable repositories, please see http://journals.plos.org/plosone/s/data-availability#loc-recommended-repositories. Any potentially identifying patient information must be fully anonymized.

Important: If there are ethical or legal restrictions to sharing your data publicly, please explain these restrictions in detail. Please see our guidelines for more information on what we consider unacceptable restrictions to publicly sharing data: http://journals.plos.org/plosone/s/data-availability#loc-unacceptable-data-access-restrictions. Note that it is not acceptable for the authors to be the sole named individuals responsible for ensuring data access.

We will update your Data Availability statement to reflect the information you provide in your cover letter.

Response: We have included the Data Availability statement: “The data used are available from the Instituto Brasileiro de Geografia e Estatística (<https://sidra.ibge.gov.br/pesquisa/ppm/quadros/brasil/2020>)” in the cover letter.

4. We note that Figures 1, 2 and 3 in your submission contain [map/satellite] images which may be copyrighted. All PLOS content is published under the Creative Commons Attribution License (CC BY 4.0), which means that the manuscript, images, and Supporting Information files will be freely available online, and any third party is permitted to access, download, copy, distribute, and use these materials in any way, even commercially, with proper attribution. For these reasons, we cannot publish previously copyrighted maps or satellite images created using proprietary data, such as Google software (Google Maps, Street View, and Earth). For more information, see our copyright guidelines: http://journals.plos.org/plosone/s/licenses-and-copyright.

We require you to either (1) present written permission from the copyright holder to publish these figures specifically under the CC BY 4.0 license, or (2) remove the figures from your submission:

a. You may seek permission from the original copyright holder of Figures 1, 2 and 3 to publish the content specifically under the CC BY 4.0 license.

We recommend that you contact the original copyright holder with the Content Permission Form (http://journals.plos.org/plosone/s/file?id=7c09/content-permission-form.pdf) and the following text:

“I request permission for the open-access journal PLOS ONE to publish XXX under the Creative Commons Attribution License (CCAL) CC BY 4.0 (http://creativecommons.org/licenses/by/4.0/). Please be aware that this license allows unrestricted use and distribution, even commercially, by third parties. Please reply and provide explicit written permission to publish XXX under a CC BY license and complete the attached form.”

Please upload the completed Content Permission Form or other proof of granted permissions as an "Other" file with your submission.

In the figure caption of the copyrighted figure, please include the following text: “Reprinted from [ref] under a CC BY license, with permission from [name of publisher], original copyright [original copyright year].”

b. If you are unable to obtain permission from the original copyright holder to publish these figures under the CC BY 4.0 license or if the copyright holder’s requirements are incompatible with the CC BY 4.0 license, please either i) remove the figure or ii) supply a replacement figure that complies with the CC BY 4.0 license. Please check copyright information on all replacement figures and update the figure caption with source information. If applicable, please specify in the figure caption text when a figure is similar but not identical to the original image and is therefore for illustrative purposes only.

The following resources for replacing copyrighted map figures may be helpful:

USGS National Map Viewer (public domain): http://viewer.nationalmap.gov/viewer/

The Gateway to Astronaut Photography of Earth (public domain): http://eol.jsc.nasa.gov/sseop/clickmap/

Maps at the CIA (public domain): https://www.cia.gov/library/publications/the-world-factbook/index.html and https://www.cia.gov/library/publications/cia-maps-publications/index.html

NASA Earth Observatory (public domain): http://earthobservatory.nasa.gov/

Landsat: http://landsat.visibleearth.nasa.gov/

USGS EROS (Earth Resources Observatory and Science (EROS) Center) (public domain): http://eros.usgs.gov/#

Natural Earth (public domain): http://www.naturalearthdata.com/

Response: The maps included in the study are not copyrighted. They were made by the authors with the assistance of QGIS software (version 3.16.13).

5. Please review your reference list to ensure that it is complete and correct. If you have cited papers that have been retracted, please include the rationale for doing so in the manuscript text, or remove these references and replace them with relevant current references. Any changes to the reference list should be mentioned in the rebuttal letter that accompanies your revised manuscript. If you need to cite a retracted article, indicate the article’s retracted status in the References list and also include a citation and full reference for the retraction notice.

Response: We have revised our reference list as recommended.

**Responses to additional Editor Comments**

1- References: there are too many congress citations. According to PLoS One guidelines, this kind of citation is only permitted if they are present online, and must have direct link (https://journals.plos.org/plosone/s/submission-guidelines)

Response: We insert the direct link or doi of the articles that were missing to insert. We use Mendeley software.

2- Figures: Please check figure axis, there is untranslated words. Please check if all figures have the complete information in English, including units and decimal.

Response: We checked the figures and made the necessary modifications as recommended.

3- Parts of the manuscript are in Portuguese:

Fig 3. Densidade do rebanho ovino por km2 132 na Amazônia Legal (a.1990, b.2000, 133 c.2010, d.2020).

Response: Thank you for your comment; we have already translated it into English.

4- Figures 1 to 3. It´s not clear what the information in the legend represents. Please clarify the information in the legend. The colors from the figures are different between, please use the same pattern, with the red color representing the higher value. At fig 3 the legend say "higher growth" but the numbers are negative?

Response: Dear Editor, we apologize because this was a mistake. But we have fixed it bying standardizing the colors of the maps with the legends.

5- Conclusion:

Despite the identification of areas with higher prevalence and potential for sheep herd growth, this activity in the Brazilian Amazon still has a low technological level, which has restricted its growth.

Considering that you used a data bank, how you can conclude that the sheep herd has low technological level? This paragraph must be moved to discussion with the inclusion or a proper reference that indicate the limited level of technology.

Response: We moved the paragraph to discussion and included some references that indicate the limited level of technology.
