# Supplementary material for: Short report: Spatial distribution and growth of sheep farming in Brazilian Amazon
Source: PLoS One. 2022 Dec 1;17(12):e0278691. doi: 10.1371/journal.pone.0278691 (PMC9714815; doi:10.1371/journal.pone.0278691)
Supplement: Attachment — Submitted filename: Response to reviewer 2.docx [file pone.0278691.s002.docx]

**Response to reviewer 2**

Reviewer #2: I have reviewed the manuscript entitled “Spatial distribution and growth of sheep farming in Brazilian Amazon” written by Andréia Santana Bezerra , Caio Cezar Ferreira de Souza, Marcos Antônio Souza dos Santos, Cyntia Meireles Martins, Maria Lúcia Bahia Lopes, Alfredo Kingo Oyama Homma , and José de Brito Lourenço Júnior and submitted to PLOS ONE as an article. The authors tried to illustrate the growth trend and its distribution of sheep in the Brazilian Amazon. Considering the limited analysis technique they used and the result they have, I suggest the authors to submit the manuscript as research communication/short communication not as a research article.

**Response**: We have made this modification, as recommended.
