# Supplementary material for: Short report: Spatial distribution and growth of sheep farming in Brazilian Amazon
Source: PLoS One. 2022 Dec 1;17(12):e0278691. doi: 10.1371/journal.pone.0278691 (PMC9714815; doi:10.1371/journal.pone.0278691)
Supplement: Attachment — Submitted filename: Response to reviewer 2_ 04-11-2022.docx [file pone.0278691.s003.docx]

Reviewer #2: My previous comments have been addressed. One minor comment I have pis the type of color used for mapping.

Red color is not a good option to indicate the occurrence of a species like sheep. We often use red color while we map a species which have a detrimental effect.

Response: Dear reviewer, we appreciate your comment, but we use the colors available in the program that formulated the maps to be able to clearly differentiate higher and lower levels of herd size and growth rate. We use the color red only to indicate the lower values of herd size and growth rate and differentiate them from the higher levels (colors ranging from dark green to yellow).
